# Notification: Artificial Intelligence in Autism Spectrum Disorder Diagnosis: A Scoping Review of Face, Voice, and Text Analysis Methods

**DOI:** 10.1002/hsr2.73405

**Published:** 2026-10-21

**NOTIFICATION**: F. Mohammadi, H. Shahrokhi, A. Asadzadeh, S. Pirmoradi, A. Moghtader, and P. Rezaei‐Hachesu, “Artificial Intelligence in Autism Spectrum Disorder Diagnosis: A Scoping Review of Face, Voice, and Text Analysis Methods,” *Health Science Reports* 8, no. 11 (2025): e71476, https://doi.org/10.1002/hsr2.71476.

This notification is for the above article, published online on 17 November 2025 in Wiley Online Library (wileyonlinelibrary.com), and has been issued by agreement between the journal Editor‐in‐Chief, Qian Chen; and John Wiley & Sons, Inc. The notification has been agreed as several articles cited in support of the section discussing AI‐based autism diagnosis through facial images have since been retracted. While readers should be informed of these developments, this does not compromise the overall conclusions of the article.

